# Pathophysiological role of prostanoids in coagulation of the portal venous system in liver cirrhosis

**DOI:** 10.1371/journal.pone.0222840

**Published:** 2019-10-23

**Authors:** Alexander Queck, Dominique Thomas, Christian Jansen, Yannick Schreiber, Sabrina Rüschenbaum, Michael Praktiknjo, Katharina Maria Schwarzkopf, Marcus Maximilian Mücke, Robert Schierwagen, Frank Erhard Uschner, Carsten Meyer, Joan Clària, Stefan Zeuzem, Gerd Geisslinger, Jonel Trebicka, Christian Markus Lange

**Affiliations:** 1 Department of Internal Medicine 1, University Hospital Frankfurt, Frankfurt, Germany; 2 Institute of Clinical Pharmacology, University Hospital Frankfurt, Frankfurt, Germany; 3 Department of Internal Medicine 1, University Hospital Bonn, Bonn, Germany; 4 Fraunhofer Institute for Molecular Biology and Applied Ecology IME, Project Group Translational Medicine and Pharmacology (TMP), Frankfurt, Germany; 5 Clinic for Gastroenterology and Hepatology, University Hospital Essen and University Duisburg-Essen, Essen, Germany; 6 Department of Radiology, University Hospital Bonn, Bonn, Germany; 7 Department of Biochemistry and Molecular Genetics, Hospital Clinic-IDIBAPS and Department of Biomedical Science, University of Barcelona, Barcelona, Spain; 8 European Foundation for the Study of Chronic Liver Failure, Barcelona, Spain; 9 Institute of Clinical Research, Odense University Hospital, University of Southern Denmark, Odense, Denmark; 10 Institute for Bioengineering of Catalonia, Barcelona, Spain; University of Navarra School of Medicine and Center for Applied Medical Research (CIMA), SPAIN

## Abstract

**Background:**

Prostanoids are important regulators of platelet aggregation and thrombotic arterial diseases. Their involvement in the development of portal vein thrombosis, frequent in decompensated liver cirrhosis, is still not investigated.

**Methods:**

Therefore, we used pro-thrombotic venous milieu generation by bare metal stent transjugular intrahepatic portosystemic shunt insertion, to study the role of prostanoids in decompensated liver cirrhosis. Here, 89 patients receiving transjugular intrahepatic portosystemic shunt insertion were included in the study, and baseline levels of thromboxane B_2_, prostaglandin D_2_ and prostaglandin E_2_ were measured in the portal and the hepatic vein.

**Results:**

While the hepatic vein contained higher levels of thromboxane B_2_ than the portal vein, levels of prostaglandin E_2_ and D_2_ were higher in the portal vein (all P<0.0001). Baseline concentrations of thromboxane B_2_ in the portal vein were independently associated with an increase of portal hepatic venous pressure gradient during short term follow-up, as an indirect sign of thrombogenic potential (multivariable P = 0.004). Moreover, severity of liver disease was inversely correlated with portal as well as hepatic vein levels of prostaglandin D_2_ and E_2_ (all P<0.0001).

**Conclusions:**

Elevated portal venous thromboxane B_2_ concentrations are possibly associated with the extent of thrombogenic potential in patients with decompensated liver cirrhosis.

**Trial registration:**

ClinicalTrials.gov identifier: NCT03584204.

## Introduction

Cirrhosis has been shown to be a pro- as well as an anticoagulatory condition[1]. Portal vein thrombosis (PVT), in particular, has been described as a sign of progression in disease stage[2]. However, factors related to PVT in cirrhosis have not been elucidated in detail.

Prostanoids are important regulators of the vascular tone, platelet aggregation and vascular remodeling. For biosynthesis of certain prostanoids, arachidonic acid is converted by cyclooxygenases (COXs) to prostaglandin (PG) G_2_ and subsequently to PGH_2_[3]. In humans, there are two COX isoforms: COX-1, expressed constitutively, and COX-2, which is inducible. PGH_2_ is subsequently converted into the prostanoids and thromboxane (TX) depending on specific synthases differentially expressed in each cell type. For example, TXA synthase (TXAS) is mainly expressed in platelets which produce TXA_2_, a lipid mediator that promotes proliferation of vascular smooth muscle cells and their constriction, platelet activation and aggregation, and finally thrombosis[4]. Importantly, COX-2 and TXAS are induced by various inflammatory stimuli, resulting in a close relationship between inflammation and regulation of the vascular tone and platelet function[5]. Hence, increased production and activity of TXA_2_ is known to contribute to the pathogenesis of diverse thrombotic arterial diseases, such as stroke or coronary heart disease[6,7]. However, little is known about the role of TXA_2_ in the portal vein and thrombosis in the portal compartment. One reason for this lack of information is that access to this compartment is difficult. Insertion of transjugular intrahepatic portosystemic stent shunt (TIPS) provides an opportunity to access the portal compartment. According to current guidelines, the major indications for TIPS insertion are treatment and prevention of variceal bleeding and/or intractable or refractory ascites[8,9]. Despite the development of polytetrafluoroethylene (PTFE) covered stents, up to 15% of patients suffer from TIPS dysfunction within the first two years[10]. Especially before the use of PTFE stents, bare metal stent insertion was associated with early TIPS dysfunction, mainly due to pro-thrombotic milieu generation. We hypothesized that prostanoids may contribute to this pro-thrombotic milieu generation. Therefore, blood samples were collected at first puncture of the portal vein during TIPS insertion and certain prostanoids, namely TXB_2_, a stable metabolite of TXA_2_, PGD_2_ and PGE_2_ were measured.

## Patients and methods

### Patients and samples

We included eighty-nine patients with diagnosed liver cirrhosis and severe portal hypertension, undergoing TIPS insertion, in our study. Primary outcome was the increase in portal hepatic venous pressure gradient (PHPG) after TIPS insertion as a read-out of pro-thrombotic milieu generation. All patients were treated between August 1996 and August 2003 in the University of Bonn, Department of Internal Medicine I, Germany. Patients, older than 18 years with clinical signs of liver cirrhosis and a multidisciplinary defined indication for TIPS insertion, were included in our trial. Exclusion criteria were the presence of systemic infection, hepatic encephalopathy (higher than grade I), bilirubinemia (higher than 5mg/dl) or arterial pulmonary hypertension. We used bare stents for TIPS insertion (8–10 mm Wallstent, Boston Scientific, MA, USA), as previously described[11,12]. Control angiography was performed after a mean of 14 days. Here, position and function of the stent was evaluated in patients with significant decrease in portal venous flow or TIPS flow (N = 64). The process flow was previously specified as routine in the center’s protocol to optimize TIPS function[13]. During the procedures, portal and hepatic venous pressures were invasively measured by the use of a pressure transducer system (Combitrans, Braun, Melsungen, Germany) and a multichannel monitor (Sirecust, Siemens, Germany). Per definition, the difference between the portal and hepatic venous pressure was named portal hepatic pressure gradient (PHPG). Once, the right branch of the portal vein was cannulated, we harvested blood from portal and hepatic vein in EDTA tubes (N = 89) to obtain material for the analyses of TXB_2_, PGD_2_ and PGE_2_. All TIPS insertions were performed without general anesthesia. After collection of the blood, we centrifuged the samples at 3000 revolutions per minute for 15 minutes at 4°C. Afterwards, plasma samples were stored at minus 80°C. Clinical patient data were recorded at their admission to the study. For the analyses of common blood parameters (e.g. creatinine or bilirubin levels), we used standard biochemical tests. The study protocol passed and has been approved by the local ethics committee of the University of Bonn (029/13). All patients signed and agreed to all procedures as declared in the study protocol. The final manuscript was reviewed by all authors. Access to the study data was ensured and all authors approved the final manuscript.

### Liquid chromatography mass spectrometry for prostanoid analysis

Two hundred μL plasma was porcupined with isotopically labeled internal standards (TXB_2_-d4, PGD_2_-d4, PGE_2_-d4), 100 μL EDTA solution (0.15M) and 600 μL ethyl acetate, to quantify levels of thromboxane B_2_, prostaglandin D_2_ and E_2_. Specimens were vortexed, and subsequently centrifuged at 20.000 g for 15 minutes. We removed the organic phase and rerun the extraction by the addition of 600 μL ethyl acetate. After combination of the organic fractions, a sparing stream of nitrogen was used for evaporation at a temperature of 45°C. Reconstitution of the residues was performed by the addition of 50 μL acetonitrile/water/formic acid (20:80:0.0025, v/v/v) and transferred to glass vials. LC-MS/MS analysis was carried out using an Agilent 1290 Infinity LC system (Agilent, Waldbronn, Germany) coupled to the hybrid triple quadrupole linear ion trap mass spectrometer QTRAP 6500+ (Sciex, Darmstadt, Germany) equipped with Turbo V source operating in negative ESI mode. The chromatographic separation was carried out using a Synergi Hydro-RP column (150 × 2 mm, 4 μm particle size and 80 Å pore size; Phenomenex, Aschaffenburg, Germany). A gradient program was employed at a flow rate of 300 μL/min. Mobile phase A was water/formic acid (100:0.0025, v/v) and mobile phase B was acetonitrile/formic acid (100:0.0025, v/v). Seperation of the analytes was performed under gradient conditions within sixteen minutes. The injection volume was 10 μL and the gradient program started with 90% A for 1 min. The mobile phase A was decreased to 60% within 1 min. After 1 min biding, the mobile phase was further decreased to 50% within 1 min and another held for 2 min. Within 2 min, mobile phase A was further decreased to 10% and held for 1 min. In the space of one minute, the initial conditions were restored. The column was re-equilibrated for 6 min. For mass spectrometric, parameters were set as follows: Source temperature 500 °C, ion spray voltage -4500 V, curtain gas 40 psi, nebulizer gas 40 psi and turbo heater gas 60 psi. Both quadrupoles were running at unit resolution. Analyst Software 1.6.3 and Multiquant Software 3.0.2 (both Sciex, Darmstadt, Germany) were used for analysis and quantification. The following precursor-to-product ion transitions were used for quantification: m/z 369.2 → m/z 195.0 for TXB_2_, m/z 351.2 → m/z 233.3 for PGD_2_ and m/z 351.2 → m/z 315.0 for PGE_2_. Peak area of the corresponding internal standard was used for correction of the peak area of each analyte. Calibration curves were constructed using linear regression with 1/x2 weighting. The coefficient of correlation was at least 0.99. Variations in accuracy were less than fifteen percent over the whole range of calibration, except for the lowest limit of quantification, where a variation in accuracy of twenty percent was accepted.

### Measurement of lipopolysaccharide levels

Using a commercial enzyme‐linked immunosorbent assay kit (Cusabio), lipopolysaccharide (LPS) serum levels were measured. Therefore, a specific antibody for LPS was precoated onto a microplate, and 100 μL of sample or standards was plated for 2 hours at room temperature. After incubation, samples were read at 450 nm. Values were expressed as picograms per milliliter; intra‐ and inter-assay coefficients of variation were 8% and 10%, respectively.

### Statistical analyses

GraphPad Prism 5 for Windows (GraphPad Software, Inc.) or BIAS® for Windows was used for the performance of statistical analyses. Wilcoxon matched-pairs test was used for paired intra-individual comparisons, namely portal versus hepatic vein prostanoid concentrations. Group differences of unrelated groups were assessed by means of χ^2^ contingency tables or Wilcoxon-Mann-Whitney U tests, as appropriate. Associations of outcomes with continuous variables were assessed in linear regression models. After univariate analyses, multivariate analyses were performed for significant associations using a P value >0.1. Variables with P >0.1 were discharged from the model. Survival analyses were performed using Cox proportional hazards regression analysis. Correlations were assessed by the use of Spearmen/Kendall rang correlation. P values < 0.05 were considered to be statistically significant.

## Results

### Baseline characteristics of patients

We included eighty-nine patients with a mean age of fifty-nine, undergoing BMS TIPS insertion, in the study. Refractory ascites (N = 41 patients; 46%) was the major indication for TIPS insertion, followed by secondary prophylaxis of variceal bleeding (N = 36 patients; 40%) and treatment of hepatorenal syndrome (N = 6 patients; 7%). In six patients (7%) indication was presence of both, esophageal variceal bleeding and ascites. Development of cirrhosis was induced in most patients by alcohol abuse (51%), followed by viral hepatitis (7%) and primary biliary cirrhosis (3%). Here, patients presented a mean MELD score of twelve points (range 6–33), and most patients were classified as Child-Pugh B at the time of study inclusion. The mean survival time after TIPS insertion was 770 days. Please find the entire patients characteristics in Table 1.

**Table 1:** Baseline characteristics of patients receiving TIPS.

Variables		Patients (n = 89)
Age (years), mean (sd)		59 (9)
Male gender, n (%)		59 (66)
Etiology of cirrhosis	alcohol, n (%)	45 (51)
	viral hepatitis, n (%)	6 (7)
	PBC, n (%)	3 (3)
	cryptogenic, n (%)	35 (39)
BMI (kg/m^2^), mean (sd)		24.7 (5.1)
Systolic blood pressure (mmHg), mean (sd)		117 (21.3)
Diastolic blood pressure (mmHg), mean (sd)		68 (10.9)
Ascites, none/ mild/ severe/ unknown, n (%)		17/ 16/ 55/ 1 (19/ 18/ 62/ 1)
Esophageal varices, grade 0/ 1/ 2/ 3/ unknown, n (%)		9/ 19/ 38/ 19/ 4 (10/ 21/ 43/ 21/ 5)
Fundus varices, grade 0/ 1/ 2/ 3/ unknown, n (%)		23/ 22/ 26/ 18 (26/ 25/ 29/ 20)
TIPS indication (bleeding/ ascites/ HRS/bleeding and ascites), n (%)		36/ 41/ 6/ 6 (40/ 46/ 7/ 7)
Hepatorenal syndrome (yes/ no/ unknown)		20/ 46/ 23 (22/ 52/ 26)
Albumin (g/dL), mean (sd)		3.1 (0.8)
Platelets (/nL), mean (sd)		124 (72.5)
Bilirubin (mg/dL), mean (sd)		1.7 (1.9)
Creatinine (mg/dL), mean (sd)		1.6 (1.2)
INR, mean (sd)		1.2 (0.2)
MELD score, mean (sd)		12 (7)
Child-Pugh class, A/ B/ C, n (%)		16/ 60/ 13 (18/ 67/ 15)
Thromboxane B_2_ (PV), pg/mL, mean (sd), median (IQR)		231.2 (621.2) 93 (131)
Prostaglandin D_2_ (PV), pg/mL, mean (sd), median (IQR)		295.6 (520.4) 88.7 (286.1)
Prostaglandin E_2_ (PV), pg/mL, mean (sd), median (IQR)		872.6 (1224.6) 473 (886.5)
Thromboxane B_2_ (HV), pg/mL, mean (sd), median (IQR)		726.9 (1709.2) 180 (552)
Prostaglandin D_2_ (HV), pg/mL, mean (sd), median (IQR)		169.4 (253) 74.8 (254)
Prostaglandin E_2_ (HV), pg/mL, mean (sd), median (IQR)		693.4 (911.6) 335 (883.6)

PBC: primary biliary cirrhosis; MELD: model of end-stage liver disease; TIPS: transjugular intrahepatic portosystemic stent shunt; INR: international normalized ratio; HRS: hepatorenal syndrome, BMI: body mass index, sd: standard deviation; PV: portal vein; HV: hepatic vein.

### Prostanoid levels and relation to patient characteristics

**Fig 1:**
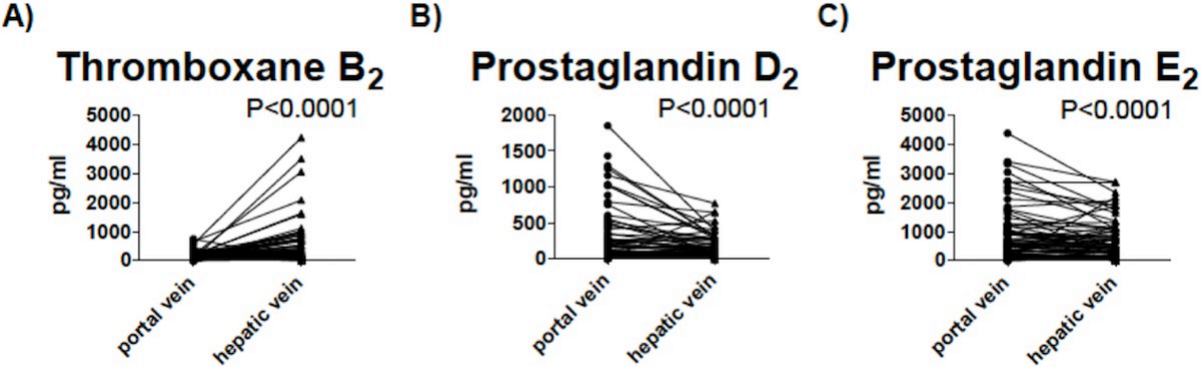
Levels of Thromboxane B_*2*_ (A), Prostaglandin D_*2*_ (B) and Prostaglandin E_*2*_ (C) in portal compared to hepatic vein. *Wilcoxon matched-pairs signed-ranks test was used to compare differences*. *P-values <0*.*05 were considered statistically significant*. *N = 89*.

Quantification of baseline TXB_2_ levels during TIPS insertion revealed significantly lower concentrations in the portal than in the hepatic vein (231.2 pg/mL vs. 726.9 pg/mL; P<0.0001). In contrast, concentrations of PGD_2_ (295.7 pg/mL vs. 169.4 pg/mL; P<0.0001) and of PGE_2_ (872.6 pg/mL vs. 693.4 pg/mL; P<0.0001) were significantly higher in the portal than in the hepatic vein (Fig 1, Table 1). Since albumin binding of prostanoids has been shown to reduce their bioavailability and activity in patients with liver cirrhosis[14], we assessed correlations of TXB_2_, PGD_2_ and PGE_2_ with serum albumin concentrations. Here, albumin concentrations correlated inversely with PGD_2_ and PGE_2_ concentrations in the portal vein (P = 0.04 each), but neither with portal vein concentrations of TXB_2_, nor with hepatic vein concentrations of TXB_2_, PGD_2_ or PGE_2_. Patient age did not affect portal or hepatic vein prostanoid levels. Correlation of white blood cell count and portal vein TXB_2_ levels revealed a significant correlation (P = 0.012). Furthermore, portal and hepatic vein levels of TXB_2_ correlated with platelet count (PV: P = 0.014; HV: P = 0.002).

The severity of liver disease (MELD score) was inversely correlated with the portal as well as the hepatic vein levels of PGD_2_ and PGE_2_ (all P<0.0001). Here, creatinine showed a significant inverse correlation with portal and hepatic vein levels of PGD_2_ and PGE_2_ and portal vein levels of TXB_2_ (Table 2).

**Table 2:** Correlation of prostanoids and different parameters.

		Albumin		WBC		Platelets		MELD score		Creatinine	
		Rho	P	Rho	P	Rho	P	Rho	P	Rho	P
**Portal vein**	TXB_2_	-0.03	0.8	**0.27**	**0.01**	**0.26**	**0.014**	-0.08	0.5	**-0.21**	**0.049**
	PGD_2_	**-0.24**	**0.038**	0.02	0.88	0.1	0.36	**-0.48**	**<0.0001**	**-0.39**	**0.0002**
	PGE_2_	**-0.23**	**0.039**	0.004	0.97	0.06	0.59	**-0.48**	**<0.0001**	**-0.37**	**0.0003**
**Hepatic vein**	TXB_2_	-0.06	0.62	0.007	0.53	**0.33**	**0.002**	-0.05	0.67	-0.16	0.14
	PGD_2_	-0.2	0.08	-0.04	0.74	0.03	0.78	**-0.47**	**<0.0001**	**-0.36**	**0.0006**
	PGE_2_	-0.16	0.15	-0.06	0.57	0.02	0.89	**-0.49**	**<0.0001**	**-0.42**	**<0.0001**

Spearman’s rank correlation test for correlation analysis. MELD: model for end-stage liver disease, WBC: white blood cell count; TXB_2_: thromboxane B_2_; PGD_2_: prostaglandin D_2_; PGE_2_: prostaglandin E_2_. P-values <0.05 were considered statistically significant.

### Association between prostanoid concentrations and portal hepatic venous pressure gradient

The mean portal hepatic venous pressure gradient (PHPG) before TIPS insertion was 20.6 mmHg, and it decreased to a mean of 9 mmHg after TIPS insertion. At control angiography, performed in 64 patients due to significant decrease in portal venous flow or TIPS flow, mean PHPG measurement was 14.6 mmHg (Table 3). Out of 64 patients, 38 had shunt dysfunction due to partial thromboses and received angioplasty. Next, uni- and multivariable regression analysis was performed to identify predictors of PHPG increase after TIPS insertion (Table 4). Of note, baseline TXB_2_ (P = 0.004) concentrations in the portal vein showed an independent correlation with PHPG increase at short time follow-up. An additional predictor of PHPG increase after TIPS insertion was baseline PHPG (P = 0.001). In contrast, no statistically significant association between portal vein concentrations of PGD_2_ and PGE_2_, or hepatic vein concentrations of TXB_2_, PGD_2_ and PGE_2_ was observed.

**Table 3:** Change of portal hepatic venous pressure gradient through TIPS-insertion over time.

Character	Before TIPS-insertion^1^ (n = 89)	P-value^1^	After TIPS insertion^2^ (n = 89)	P-value^2^	Follow-up angiography^3^ (n = 64)	P-value^3^
PHPG, mean	20.6	**<0.0001**	9	**<0.0001**	14.6	**<0.0001**
1–10 mmHg, n	0		60		16	
11–16 mmHg, n	14		25		28	
17–20 mmHg, n	33		3		10	
21–25 mmHg, n	32		1		9	
>25 mmHg, n	10		0		1	

Patient characteristics at time point 1 (baseline at insertion of TIPS), time point 2 (directly after TIPS insertion) and time point 3 (Follow-up angiography); TIPS: transjugular intrahepatic portosystemic stent shunt; PHPG: portal hepatic venous pressure gradient. P-values <0.05 were considered statistically significant.

1 between time point 1 and 2

2 between time point 2 and 3

3 between time point 1 and 3.

**Table 4:** Regression analysis of factors associated with increase of PHPG in control angiography compared to baseline after TIPS-insertion.

	univariable model		multivariable model	
	β	P-value	β	P-value
TXB_2_ (PV)	**0.003**	**0.004**	**0.003**	**0.004**
PGD_2_ (PV)	0.008	0.16		
PGE_2_ (PV)	-0.005	0.08	-0.014	0.15
TXB_2_ (HV)	0.0002	0.7		
PGD_2_ (HV)	0.02	0.2		
PGE_2_ (HV)	-0.0003	0.9		
Portal hepatic venous pressure gradient	**-0.54**	**0.002**	**-0.56**	**0.001**

Multivariable linear regression analysis. TXB_2_: thromboxane B_2_; PGD_2_: prostaglandin D_2_; PGE_2_: prostaglandin E_2_; PV: portal vein; HV: hepatic vein; P-values <0.05 were considered statistically significant. N = 64 patients

In a subset of 17 patients, we analyzed portal vein levels of lipopolysaccharide (mean 57.4 pg/ml +/- 9.6pg/ml). Spearmen’s rank correlation did not show significant correlations between portal vein levels of lipopolysaccharide and portal vein levels of PGD_2_ (R = -0.26; P = 0.31), PGE_2_ (R = -0.23; P = 0.37), or TXB_2_ (R = -0.02; P = 0.95).

### Predictors of survival after TIPS insertion

COX regression analysis of survival after TIPS insertion revealed baseline creatinine (P<0.0001) concentrations as a negative predictor of survival (S1 Table). In detail, median survival of patients with baseline creatinine concentration ≥1.2 mg/dL was 236 days compared to 820 days in patients with creatinine concentration <1.2 mg/dL (P = 0.0005)(S1 Fig). However, no significant association between prostanoid concentrations in the portal or hepatic vein and patient survival after TIPS insertion was observed.

## Discussion

The major finding of the present study is that concentrations of TXB_2_ are associated with an increase of PHPG as an indirect sign of thrombogenic potential.

Portal hypertension (PHT) is associated with sequelae, such as ascites, variceal bleeding and hepatorenal syndrome. Besides aggravation of portal hypertension due to cirrhosis, PHT can also be impaired by the development of portal vein thrombosis, which frequently occurs in cirrhosis. These mechanisms are not fully understood. To the best of our knowledge there are no correlations of prevalence of thrombosis with portal pressure. Therefore, this study delivers evidence for a potential mechanism leading to portal hypertension, cirrhosis and PVT.

TXA_2_ is a potent vasoconstrictor and a major activator of platelets synthesized by TXAS from PGH_2_, a downstream metabolite of arachidonic acid which is preferentially catalyzed by constitutively expressed COX-1. In turn, TXA_2_ is highly unstable and rapidly converted into its inactive hydrolysis product TXB_2_, which is a good surrogate marker of TXAS activity and TXA_2_ levels. The observation of higher TXB_2_ concentrations in the hepatic vein compared to the portal vein might be explained by the abundance of COX-1 and COX-2 in liver sinusoidal epithelial cells, which was observed in an animal model of liver cirrhosis[15]. Moreover, high levels of phospholipase A2, an enzyme needed to catalyze the release of arachidonic acid from membrane phospholipids, were observed in the cirrhotic liver of rats[16,17]. However, the experimental evidence about inducible TXB_2_ release by Kupffer cells in fibrosis with a consequent increase of portal pressure[18] supports our recent finding of elevated TXB_2_ levels in the human hepatic vein in decompensated liver cirrhosis. At least in animal models, the distribution of these enzymes results in high levels of hepatic TXB_2_, which is in line with our finding of a post-hepatic increase of TXB_2_ concentrations[19]. Indeed, aggregation of platelets in cirrhosis might lead to obliteration and parenchymal extinction, which is associated with portal hypertension[20]. This observation was confirmed by the correlation between platelets and TXB_2_. Also, in the portal vein, TXB_2_ seems to play a specific role, since it is independently associated with an increase of PHPG and correlated with the white blood count. While in our study, TXB_2_ concentrations in the portal vein were lower than in the hepatic vein, they must be considered as high, since significantly lower TXB_2_ concentrations were observed in the systemic circulation of healthy individuals (no data on portal venous TXB_2_ concentrations have become available to date)[21,22]. Another possible explanation of the TXB_2_ correlation with increase in PHPG is that TXB_2_ induced contraction of hepatic stellate cells[23].

Hence, the association observed here between TXB_2_ concentrations in the portal vein and increase in PHPG after TIPS insertion appears to be biologically plausible. Furthermore, one may speculate that high levels of TXB_2_ in the portal vein possibly predispose to the aggravation of PHT and TIPS dysfunction, particularly since it is clearly linked to inflammation, as suggested by the white blood count correlation.

In contrast to TXB_2_, concentrations of PGE_2_ and PGD_2_ were higher in the portal than in the hepatic vein. While we can only speculate on the functional basis of this observation, it is known that the enzymes generating PGD_2_[24] and PGE_2_[25] are widely distributed in various tissues and, in particular, in inflammatory cells. Therefore, it appears plausible that the high concentrations of PGD_2_ and PGE_2_ in the portal vein originate from bacterial translocation, a hallmark of decompensated liver cirrhosis[26,27]. Although PGE_2_ has rather minor effects on platelets, PGE_2_ effects on the vascular tone are the opposite of those of TXA_2_, as PGE_2_ signaling results in vasodilation[28]. The inverse association, even if not statistically significant, between PGE_2_ concentration in the portal vein and increase of PHPG after TIPS insertion further supports the potential pathophysiological relevance of our findings.

Interestingly, portal and hepatic vein levels of PGD_2_ and PGE_2_ were inversely correlated to the severity of liver diseases as determined by the MELD score. While it is known that PGD_2_ and PGE_2_ are secreted by inflammatory cells, here, we did not find a correlation with the white blood count. This can be explained either by immune paralysis due to advanced liver disease with reduced PGD_2_- and PGE_2_ release by inflammatory cells[29] or the possibility that other cells are the main producers of PGD_2_ and PGE_2_ in this pathological setting.

Evaluating inhibitors of prostanoid synthesis, namely of the TXA_2_-pathway, for prevention of portal vein thrombosis will be of interest here. These anti-platelet agents not only have anti-thrombotic functions in liver cirrhosis, but also anti-fibrotic and chemoprevention effects inhibiting hepatocellular carcinoma in animal models of liver disease and in clinical association studies[30–33]. On the one hand, non-steroidal anti-inflammatory drugs (NSAID) decrease concentrations of TXA_2_ by inhibition of COX and may reduce vascular tone in the liver. On the other hand, administration of NSAIDs reduces renal blood flow as well as the glomerular filtration rate in cirrhotic patients with ascites and induces renal failure[34–36]. Moreover, the equilibrium between thrombosis and bleeding is very unstable in liver cirrhosis. Therefore, one should be very careful with systemic drugs in cirrhosis, especially with COX inhibition. Therefore, systemic administration of COX inhibitors is obsolete in cirrhosis and ascites[9]. Since in animal models, COX inhibition seems to be useful in terms of portal hypertension, this liver-specific approach may be considered, as previously shown for Rho-kinase inhibition[37].

Our study has limitations. We were unable to include a control group of healthy individuals, because no medical interventions allowing for withdrawal of portal or hepatic venous blood were performed in these individuals. Unfortunately, the comparison of peripheral prostanoid levels of healthy controls, compensated and decompensated cirrhotic patients was not focus of this study. Furthermore, we quantified total prostanoid concentrations in the knowledge that albumin binding may also play a role in their bioavailability and activity, in particular in patients with decompensated liver cirrhosis[14]. However, similar results were found when PGD_2_ and PGE_2_ were normalized to the respective albumin levels in our study. Moreover, we focused only on the stent thrombosis and other territories were not evaluated. Finally, we can’t exclude the role of the bile leakage on the generation of the thrombogenic milieu and a possible interaction with the prostanoids. Future studies may address all these limitations.

## Conclusion

In conclusion, elevated portal venous TXB_2_ concentrations are possibly associated with the extent of thrombogenic potential in patients with decompensated liver cirrhosis and offer suitable targets for future therapies.

## Supporting information

S1 Table Cox regression analysis of factors predicting mortality of patients receiving TIPS. (DOCX)

S1 Fig Survival after TIPS insertion in dependency of baseline serum creatinine levels. (TIF)

## Abbreviations

COX: cyclooxygenase
HV: hepatic vein
PGD_2_: prostaglandin D_2_
PGE_2_: prostaglandin E_2_
PHPG: portal hepatic venous pressure gradient
PV: portal vein
PVT: portal vein thrombosis
TXA_2_: thromboxane A_2_
TXB_2_: thromboxane B_2_
