# Supplementary material for: Pathophysiological role of prostanoids in coagulation of the portal venous system in liver cirrhosis
Source: PLoS One. 2019 Oct 23;14(10):e0222840. doi: 10.1371/journal.pone.0222840 (PMC6808498; doi:10.1371/journal.pone.0222840)
Supplement: S1 Table — (DOCX) [file pone.0222840.s001.docx]

**Supplementary information:**

**SI-Table 1: Cox regression analysis of factors predicting mortality of patients receiving TIPS.**

| **Relative Hazard Ratio (exp-β) and 95%- CI** | | | | |
| --- | --- | --- | --- | --- |
| **univariate model** | | | **multivariate model** | |
| Variables | HR (95% CI) | P-value | HR (95% CI) | P-value |
| **Creatinine** | **1.55 (1.27-1.88)** | **<0.0001** | **1.5 (1.24-1.8)** | **<0.0001** |
| TXB_2_ (portal vein) | 1 (1-1.01) | 0.25 |  | |
| PGD_2_ (portal vein) | 1.01 (1-1.01) | 0.36 |  |  |
| PGE_2_ (portal vein) | 1 (1-1.01) | 0.31 |  |  |
| TXB_2_ (hepatic vein) | 1 (1-1.01) | 0.66 |  |  |
| PGD_2_ (hepatic vein) | 1 (1-1.01) | 0.90 |  |  |
| PGE_2_ (hepatic vein) | 1 (1-1.01) | 0.58 |  |  |

Multivariate linear regression analysis. CI: confidence interval; TIPS: transjugular intrahepatic portosystemic stent shunt; TXB_2_: thromboxane B_2_; PGD_2_: prostaglandin D_2_; PGE_2_: prostaglandin E_2_; P-values <0.05 were considered statistically significant. N=89 patients.
