# Supplementary figures and images for: Pathophysiological role of prostanoids in coagulation of the portal venous system in liver cirrhosis
Source: PLoS One. 2019 Oct 23;14(10):e0222840. doi: 10.1371/journal.pone.0222840 (PMC6808498; doi:10.1371/journal.pone.0222840)

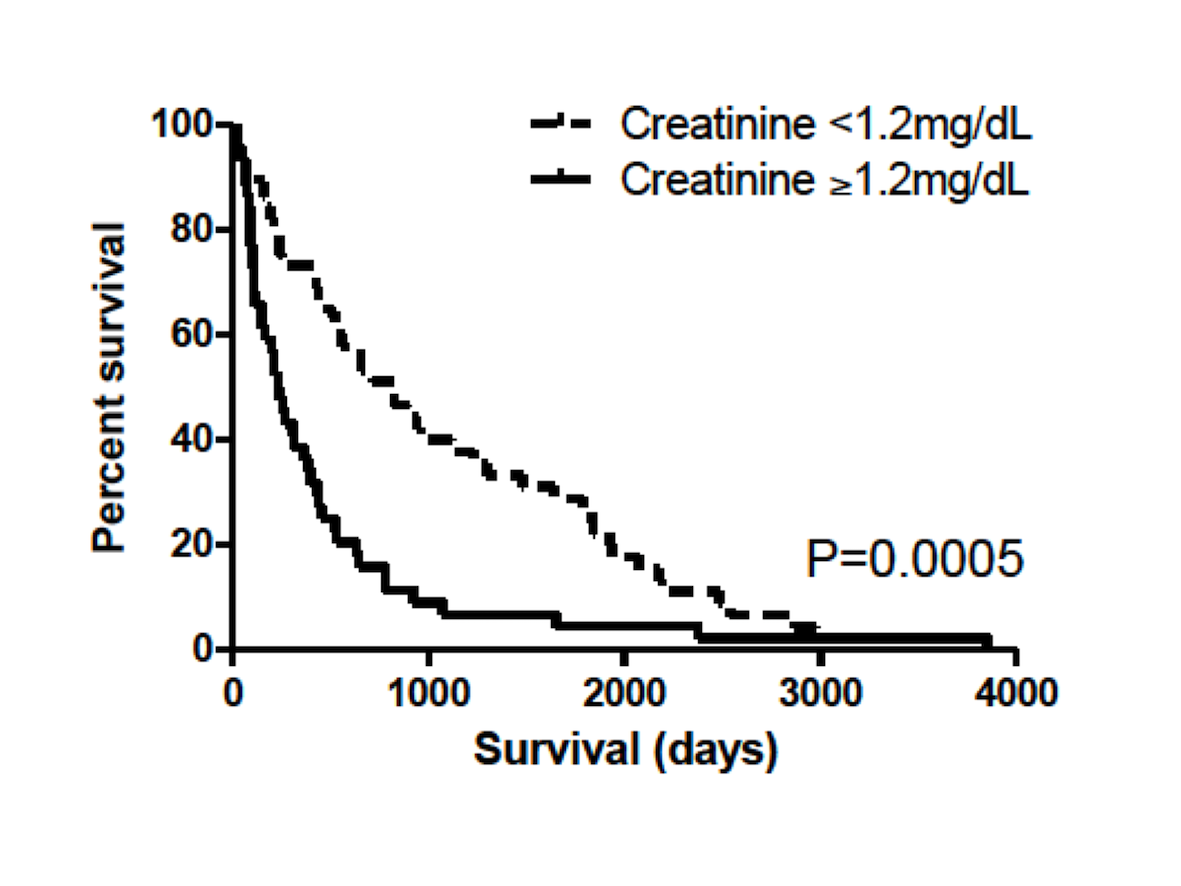

Supplement: S1 Fig — (TIF) [file pone.0222840.s002.tif]
